# A Modified Ceramic-Coating Separator with High-Temperature Stability for Lithium-Ion Battery

**DOI:** 10.3390/polym9050159

**Published:** 2017-04-29

**Authors:** Chuan Shi, Jianhui Dai, Chao Li, Xiu Shen, Longqing Peng, Peng Zhang, Dezhi Wu, Daoheng Sun, Jinbao Zhao

**Affiliations:** 1 Industrial Research Institute of nonwovens & Technical Textiles, College of Textiles & Clothing, Qingdao University, 266071 Qingdao, China; chuanshi@qdu.edu.cn; 2 College of Chemistry and Chemical Engineering, State key laboratory of Physical Chemistry of Solid Surfaces, Collaborative Innovative Center of Chemistry for Energy Materials, Xiamen University, 361005 Xiamen, China; leechao@stu.xmu.edu.cn (C.L.); xiushen@stu.xmu.edu.cn (X.S.); 20520151152394@stu.xmu.edu.cn (L.P.); 3 College of Energy Research & School of Energy Research, Xiamen University, Xiamen 361102, China; daijh1992@stu.xmu.edu.cn (J.D.); pengzhang@xmu.edu.cn (P.Z.); 4 School of Aerospace Engineering, Xiamen University, Xiamen 361005, China; wdz@xmu.edu.cn (D.W.); daohs@xmu.edu.cn (D.S.)

**Keywords:** lithium-ion battery, separator, polydopamine, ceramic coating, high safety

## Abstract

In this work, the ceramic coating separator (CCS-CS) prepared with polyethylene (PE) separator, Al_2_O_3_ inorganic particles, carboxymethyl cellulose sodium (CMC) and styrene-butadiene rubber (SBR) mix binders is further modified by coating with a thin polydopamine (PDA) layer through a simple chemical deposition method. Compared with the bare ceramic coating separator, the PDA-modified CCS-CS (CCS-CS-PDA) exhibits excellent thermal stability, which shows no thermal shrinkage after storing at 200 °C for 30 min. Compared with the PE separator, both the uptake and wettability with the electrolyte and water of CCS-CS-PDA are improved significantly. Meanwhile, when saturated with liquid electrolyte, the CCS-CS-PDA also shows enabled high ionic conductance. Furthermore, the test of the electrochemical impedances changing with the temperatures suggests that only the PE separator exhibits no thermal shutdown behaviors, and the CCS-CS separator only has a shutdown temperature range from 138 to 160 °C, while the CCS-CS-PDA shows a shutdown temperature range from 138 to more than 200 °C. The cells prepared with the CCS-CS-PDA also show stable repeated cycling performance and good rate capacity at room temperature.

## 1. Introduction

In recent decades, polypropylene (PP) and polyethylene (PE) porous membranes (polyolefin separator) with good mechanical strength and excellent chemical stability have been used as separators for assembling lithium-ion batteries [1,2]. Especially, the multilayer polyolefin separator as PP/PE/PP with the shutdown function is commonly used as a fail-safe device in commercial cells [1,3]. However, both extremely low wettability with liquid electrolytes and weak thermal stability have limited its further application in energy storage systems especially in hybrid electric vehicles (HEVs) areas [4,5,6]. To remedy the above defects, different kinds of separators were reported, such as polymers’ surface grafting modification of polyolefin separators [7,8,9], ceramic coating separators [6,10,11,12,13,14], nonwoven membrane [15,16,17,18,19,20] and polymer electrolyte used as separators [21,22,23,24,25,26].

Among the above methods, the ceramic coating separators (CCS) prepared by coating inorganic materials onto the surface of pure supporting separators improve the thermal stability and wettability of the polyolefin separators well, meanwhile retaining the good characteristics of basic separators. However, the CCS still show obvious thermal shrinkage at high temperatures [27,28,29]. Therefore, they still cannot meet the requirements of high safety batteries. Ryou et al. have reported a surface modification method using PDA for polyolefin separators [30,31,32,33,34,35]. It has been proven that the separator matrix will not be harmed after coating with the PDA layer [36], and the PDA-coated separators usually show good mechanical and electrochemical properties and perform well in lithium-ion batteries [32,33]. In our previous work, to further improve the thermal stability of CCS, it was modified by coating with a thin polydopamine (PDA) layer through a simple chemical deposition method. Combining the function of the PE separator, SiO_2_ inorganic particles and PDA layers, the developed composite-modified separator displays substantially enhanced thermal and mechanical stability, with no visual thermal shrink and can maintain its mechanical strength up to 230 °C [29]. Compared with one-side coating, the double side coating method can ensure the uniformity of the separator, then the thermal and mechanical stability of the separator will be improved. However, the two processes of double side coating will be influenced by each other. The homogeneity of the CCS is difficult to control, especially in the actual industrial product process. What is more, double side coating costs much more than one side coating, combined with the fact that the SiO_2_ particles with regular morphology production are low. This resulted in the practical application of the modified separator being limited.

Aiming to solve the above problems, meanwhile maintaining the thermal stability of the separator, in this work, the Al_2_O_3_ particles (mass production) were selected as the coating materials, and the basic CCS was prepared with the one side coating method. Then, the surface of CCS was further coated with a thin layer of PDA by the chemical deposition method, and the deposition time was adjusted to guarantee the thermal stability of the separator. Compared with the use of SiO_2_ spherical particles and double side coating, the use of Al_2_O_3_ inorganic particles and one side coating method is more suitable for large-scale industrialization. The PDA-modified CCS-CS (CCS-CS-PDA) exhibited excellent thermal stability, which showed no thermal shrinkage after storing at 200 °C for 30 min. The test of the electrochemical impedances changing with the temperatures suggested that only the PE separator exhibited no thermal shutdown behaviors, and the CCS-CS separator only had a shutdown temperature ranging from 138 to 160 °C, while the CCS-CS-PDA showed a shutdown temperature range from 138 to more than 200 °C. The cells prepared with the CCS-CS-PDA also showed stable repeated cycling performance and rate capacity at room temperature. Therefore, such advantages of CCS-CS-PDA mentioned above will make it suitable for practical applications in secondary lithium batteries, especially in hybrid electric vehicles (HEVs) and energy storage systems areas. 

## 2. Experiments

### 2.1. Fabrication of the CCS-CS and CCS-CS-PDA

The PE separator (thickness of 20 μm, Asahi Kasei, Tokyo, Japan) was used as a support layer. Deionized water- and ethyl alcohol-based (5 mL:5 mL) coating solution containing 0.95 g Al_2_O_3_ (average particle size of 400 nm, Taimei Chemicals, Nagano, Japan), 0.03 g SBR and 0.02 g carboxymethyl cellulose sodium (CMC) (Guangzhou Songbai Chemical, Guangzhou, China) was prepared for CCS-CS. The detailed preparation process of CCS-CS can be found in our earlier works [27]. The thickness of the coating layer was accurately controlled at 4 μm.

The aqueous/ethanol-based (equal volume) solution, consisting of 2 mg dopamine hydrochloride (Sigma Aldrich, MI, USA) per milliliter and 10 mM tris(hydroxymethyl)aminomethane with a pH of 8.5, was used for the preparation of CCS-CS-PDA. After simple immersion of the CCS-CS in the above solution for 48 h, a thin PDA layer will spontaneously deposit on the surface of CCS-CS. The modified ceramic coating separator (CCS-CS-PDA) was dried under a vacuum line at 80 °C over night to remove the residual solvent.

### 2.2. Electrode Preparation and Cell Assembly

Coin cells were prepared for the batteries’ performance tests. A mixture of slurry containing 90 wt % LiMn_2_O_4_ (Qingdao Xinzheng Material Co., Ltd., Qingdao, China), 5 wt % acetylene black (super-P) and 5 wt % polyvinylidene fluoride (PVDF) in *N*-methyl pyrrolidine (NMP) was prepared for the cathode of the cells. The PE separator, CCS-CS and CCS-CS-PDA were used as separators for preparing the batteries. Batteries after injecting the same weight of electrolyte (1 mol·L^–1^ LiPF_6_ dissolved in a mixed solution of ethylene carbonate (EC), dimethyl carbonate (DMC) and diethyl carbonate (DEC) with a volume ratio of 1:1:1) were assembled in argon gas with a glove box (Mbraun, Munich, Germany).

### 2.3. Characterization of the Separators

The surface and cross-section morphologies (the separators were broken mechanically after being cooled in liquid nitrogen for the cross-section morphologies’ measurement) of the pure PE separator, CCS-CS and CCS-CS-PDA were investigated by a field-emission scanning electron microscope (FE-SEM, S4800, Hitachi, Tokyo, Japan) at an acceleration voltage of 15 kV.

The porosity changes of the CCS-CS before and after PDA coating and of the separators under different temperatures can be calculated as the following equation with the *n*-butanol uptake method:

(1) $$P(\%)=M_{\mathrm{BuOH}}/(\rho_{\mathrm{BuOH}}\times (M_{\mathrm{BuOH}}/\rho_{\mathrm{BuOH}}+M_{m}/\rho_{p}))\times 100\%$$

where *ρ*_P_ and *ρ*_BuOH_ represent the densities of polymer and *n*-butanol, while *M*_m_ and *M*_BuOH_ represent the mass of the membrane before and after absorbing *n*-butanol, respectively.

The thermal stability of the PE separator, CCS-CS and CCS-CS-PDA (original size: 2 cm × 2 cm) was investigated by measuring their dimensional change using the following equation after subjecting them to various temperatures from 110 to 200 °C for 0.5 h:

(2) $$\mathrm{Shrinkage}\%=\frac{S_{0}-S}{S_{0}}\times 100\%$$

where *S*_0_ and *S* are the areas of the membranes before and after the heat-treating test, respectively.

The static contact angle of the PE separator, CCS-CS and CCS-CS-PDA with the electrolyte and deionized water was taken by using a commercial drop shape analysis system (Powereach JC2000C1, Shanghai Zhongchen Digital Technique Equipment Co. Ltd., Shanghai, China). The electrolyte uptake of the membranes was calculated by the following equation:

(3) $$\mathrm{Uptake}\%=\frac{W-W_{0}}{W_{0}}\times 100\%$$

where *W*_0_ and *W* are the weights of the membranes before and after absorbing the liquid electrolyte, respectively. 

The ionic conductivities (impedance data) of the PE separator, CCS-CS and CCS-CS-PDA absorbed liquid electrolyte and sandwiched between two stainless steel electrodes were investigated by electrochemical workstation (Solartron, SI-1260, West Sussex, UK) with the frequency range of 1 Hz to 100 kHz. The shutdown behaviors of the PE separator, CCS-CS and CCS-CS-PDA were investigated by heating the separators at the rate of 1 °C·min^–1^ and recording the impedance data.

The cells with CCS-CS and CCS-CS-PDA were prepared to investigate the influence of the modified PDA layer on the cycle and rate capability at the electrochemical test equipment (LAND-V34, Land Electronic, Wuhan, China). To study the cycling performances of the batteries, the cells were charged to 4.2 V and discharged to 3 V at 1.0 C, and the rate performances were carried out at current rates of 0.5, 1.0, 2.0, 4.0 and 0.5 C.

## 3. Results and Discussion

**Figure 1:**
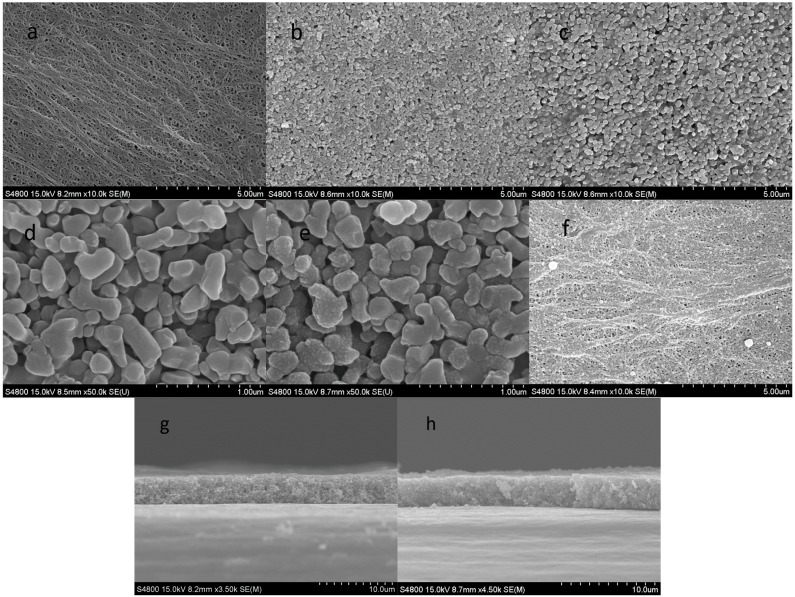
SEM morphology: (**a**) PE, (**b**,**d**) CCS-CS, (**c**,**e**) CCS-CS-PDA and (**f**) PE side of CCS-CS-PDA. Cross-section morphology: (**g**) CCS-CS and (**h**) CCS-CS-PDA.

The top surface and cross-section scanning electron micrographs of the pristine PE, CCS-CS and CCS-CS-PDA membranes are comparatively displayed in Figure 1. The bare PE separator (with a thickness of 20 μm) shows an interconnected submicron porous structure, which is the typical morphology of the wet process (Figure 1a). After coating with inorganic particles, the separator surfaces were homogeneously covered with Al_2_O_3_ particle and the CMC and SBR mix binders shown in Figure 1b. Compared with the CCS-CS, there was no significant morphology change of the CCS-CS-PDA shown in Figure 1c, except that the rough dopamine-coated surface replaced the smooth surface of the Al_2_O_3_ (shown in Figure 1d,e). The PE surface of the CCS-CS-PDA was uniformly covered by compact PDA layers as shown in Figure 1f. Moreover, there were also some self-polymerization PDA particles deposited on the PE surface during the modified process. Figure 1g,h shows the cross-sectional views of CCS-CS and CCS-CS-PDA. The figures clearly show that the PDA coating process did not increase the thickness of the coating layer (about 4 μm). 

The weight and porosity changes of CCS-CS before and after PDA modification were measured and summarized in Table 1. The weight of CCS-CS increased from 4.3 to 4.6 mg (per a diameter of 1.85 cm of the wafer), meanwhile, the porosity of the CCS-CS decreased from ~41% to ~36%. Both changes mentioned above with the results of changes in the morphologies all together corroborated that the CCS-CS-PDA was prepared successfully.

The separator is used to prevent the contact of the cathode with the anode and meanwhile to provide conducting channels for lithium ions when soaked in electrolyte [1]. However, the conventional polyolefin separators easily suffer thermal shrinkage under high temperatures as they are fabricated by multiple stretching processes and due to their low melt temperatures [4,37]. Our previous work has proven that the ceramic coating can make the polyolefin separators resistant against thermal shrinkage due to the high thermal stability of Al_2_O_3_ and CMC and SBR binders [27]. In this study, the thermotolerance is supposed to be further enhanced after the coating thermal stability PDA layer on the surface of the ceramic Al_2_O_3_ coating layer.

**Figure 2:**
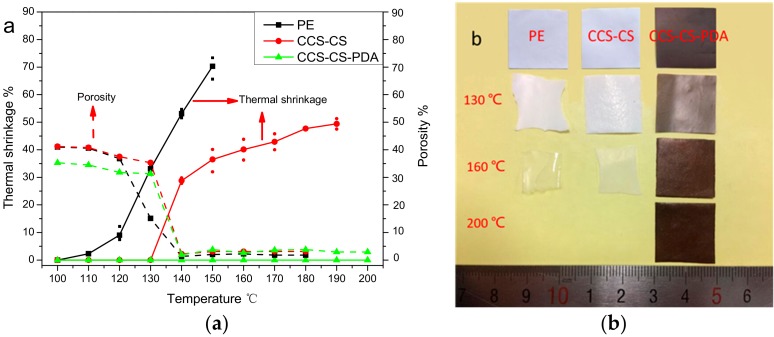
(**a**) Thermal shrinkage and porosity (%) changes of the CCS-CS and CCS-CS-PDA membrane. (**b**) Photograph of the PE, CCS-CS and CCS-CS-PDA after being held at different temperatures for 30 min.

Thermal shrinkage properties of the PE separator, CCS-CS and CCS-CS-PDA were quantitatively investigated by measuring the area changes after being subjected to various temperatures from 110 to 200 °C for 30 min, and the results are shown in Figure 2a. The pure PE separator exhibited a significant thermal shrinkage ratio of 8% after being stored at 110 °C for 30 min, while there were no area changes for both the CCS-CS and CCS-CS-PDA separators. The pure PE separator originated from a uniaxial stretching method, such that the productive process for the PE separator had to undergo the thermal shrinkage at 110 °C (the thermal shrinkage of the PE separator occurred far below the melting temperature of PE materials) [1,27]. With the increase of the test temperatures, about 30% thermal shrinkage of CCS-CS occurred after being stored at 140 °C for 30 min. The thermal shrinkage of the PE separator and CCS-CS increased with the increasing temperatures while the CCS-CS-PDA suffered no thermal shrinkage, even at 200 °C. Shrinkage under high temperatures can be effectively suppressed after coating the PE separator with heat-resistant Al_2_O_3_ inorganic particles and CMC and SBR mix binders. However, the CCS-CS still showed obvious thermal shrinkage at high temperatures above 140 °C. The CCS-CS-PDA showed higher thermal stability than the PE separator and CCS-CS at a wider range of temperatures. This improvement in thermal stability of the CCS-CS-PDA can be attributed to the use of Al_2_O_3_, CMC and SBR mix binders and PDA. The melting temperature of the PDA is above 230 °C, resulting in its better ability to suppress the shrinkage of the CCS-CS-PDA than the CCS-CS at elevated high temperatures. The shrinkage of CCS-CS can be effectively suppressed after being modified with heat-resistant PDA, which is particularly important for the high-capacity lithium-ion batteries prepared for HEVs and energy storage systems.

Photographs of the PE, CCS-CS and CCS-CS-PDA after being held at 130, 160 and 200 °C, respectively, for 30 min are shown in Figure 2b. The photographs clearly exhibit that the PE separator suffered a large percent of thermal shrinkage, while both CCS-CS and CCS-CS-PDA separators stayed stable after being stored at 130 °C for 30 min. With further increases of the test temperatures, both the PE separator and CCS-CS presented significant thermal shrinkage and a clear color change from white to transparent at 160 °C. On the contrary, CCS-CS-PDA still remained intact even after being stored at 200 °C for 30 min. The above difference is due to the application of the PDA coating layer [32,33].

The porosity variations with temperatures were measured and are shown in Figure 2a. The porosities of the PE separator and CCS-CS are almost the same before the heat treatment, which suggests that the porosities of the ceramic coating layer and PE separator are very close. After PDA modification, the porosity of the CCS-CS decreased as expected. The porosities of the PE separator, CCS-CS and CCS-CS-PDA all were relatively stable before 130 °C. However, the porosity of the PE separator dropped rapidly after heat treatment at 130 °C for 30 min, while the porosities of CCS-CS and CCS-CS-PDA just decreased slightly. The above results coincide with the results of the thermal shrinkage test. With the further increase of the test temperatures, the porosities of the PE separator, CCS-CS and CCS-CS-PDA all dropped rapidly at above 140 °C, which was caused by the shutting of holes inside the PE separator. Although there are measurement errors, the porosities of the CCS-CS-PDA, CCS-CS and PE separator are in descending order after the heat treatment test at above 140 °C, as shown in Figure S1.

**Figure 3:**
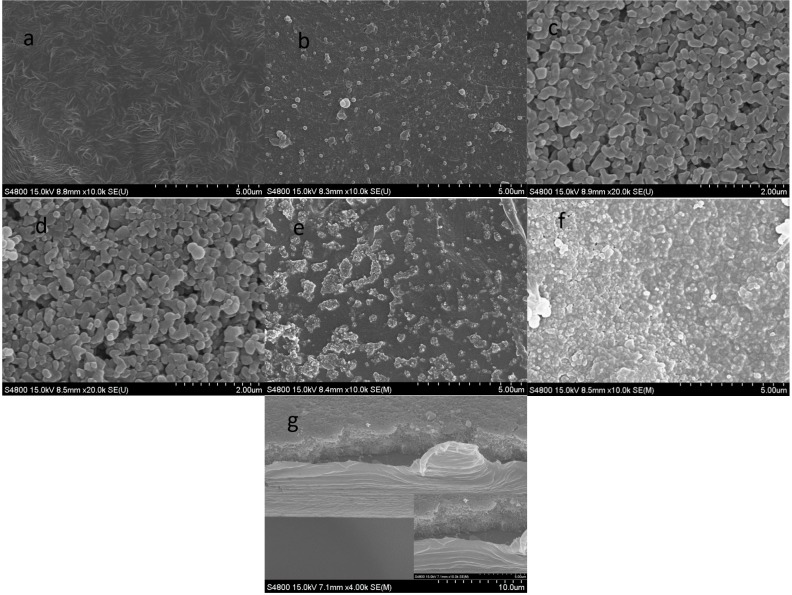
The SEM morphologies after 150 °C treatment: (**a**) the PE side of CCS-CS; (**b**) the PE side of CCS-CS-PDA; (**c**) the coating side of CCS-CS; (**d**) the coating side of CCS-CS-PDA. The SEM morphologies after 200 °C treatment: (**e**) the coating side of CCS-CS; (**f**) the coating side of CCS-CS-PDA; (**g**) the cross-section of CCS-CS-PDA.

To further study the causal factors for the porosity change of the separator, the SEM images of the CCS-CS and CCS-CS-PDA after annealing treatment were observed and are shown in Figure 3. It can be seen from Figure 3a,b that the porous PE surfaces of CCS-CS and CCS-CS-PDA became a smooth surface (with no holes on the surface). The ceramic coating surfaces of CCS-CS and CCS-CS-PDA exhibited no obvious change after heat treatment at 150 °C for 30 min. However, with the further increase of the test temperature to 200 °C, the PE support layer and ceramic coating layer of CCS-CS melted together, as shown in Figure 3e, while the ceramic coating layer of CCS-CS-PDA kept stable, and parts of PE melted and flowed into the gap among the Al_2_O_3_ particles, as shown in Figure 3f,g. Moreover, PE melted and flowed into the gap among the Al_2_O_3_ particles combined with the shutting of the holes inside the PE separator, resulting in a thickness decrease of the PE support layer. The above result coincided with the porosity change of the separator at high temperature.

**Figure 4:**
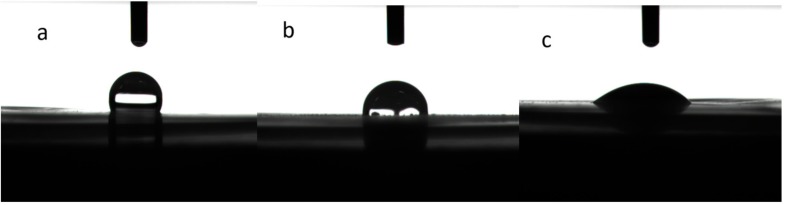
Contact angle tests: (**a**) PE separator, (**b**) CCS-CS, (**c**) CCS-CS-PDA.

As mentioned above, the polyolefin-based separators inherently suffer from poor wetting with polar liquid electrolytes [38]. The poor wettability of the separator with the electrolyte can increase the electrolyte filling time during the assembly process and impact the ability to retain the electrolyte solution, thereby affecting the performance of the battery. Coating with Al_2_O_3_ and PDA can help improve the wetting ability of the PE separator [27,35]. To investigate the effect of the ceramic and PDA coatings on the wetting abilities of the separators, the contact angles of liquid electrolyte on the surface of the membranes were measured and are summarized in Table 1. The PE separator showed a contact angle of about 35.0°, which was much higher than that for the CCS-CS (0°) and CCS-CS-PDA (0°). Such low contact angles of CCS-CS and CCS-CS-PDA represented excellent wettability of the surface with the electrolyte. In order to further distinguish the wettability of CCS-CS and CCS-CS-PDA, the contact angle measurements were conducted by water droplet, and the results were shown in Figure 4. The PE separator showed a contact angle about 110.0°, which was much higher than that of the CCS-CS (85°) and CCS-CS-PDA (35°) after the same contact time of the separator interface with the deionized water. The result suggested that the wettability of CCS-CS was further improved after surface-modification with PDA.

Since the only media to transport the lithium ion is the separator that absorbed the electrolyte, the electrolyte uptake of separators as an important characteristic was measured and is summarized in Table 1. The electrolyte uptakes of the PE, the CCS-CS and the CCS-CS-PDA are 54.0%, 71.2% and 70.3%, respectively. The electrolyte uptake capacity improvement of CCS-CS and CCS-CS-PDA can be contributed to by the increased wettability since the porosity of the PE support layer and ceramic coating layer is close. The electrolyte can be easily be trapped onto the ceramic coated and PDA-modified surface and extended into the inner pore of the separator. The electrolyte uptake of the CCS-CS-PDA is a little lower than the CCS-CS because of the interaction of decreased porosity and improved wettability. 

The effects of the PDA coating layer on the ionic conductivities of membranes were also studied, and the results were shown in Table 1. The ionic conductivities of the PE separator, CCS-CS and CCS-CS-PDA after being soaked in electrolyte solution are 0.78, 1.10 and 0.71 mS·cm^–1^ at 20 °C, respectively. The ionic conductivities of CCS-CS-PDA and the PE separator are close, but lower than the CCS-CS. The porosity decrease after PDA coating is the main reason for the ionic conductivities decrease of CCS-CS-PDA.

**Figure 5:**
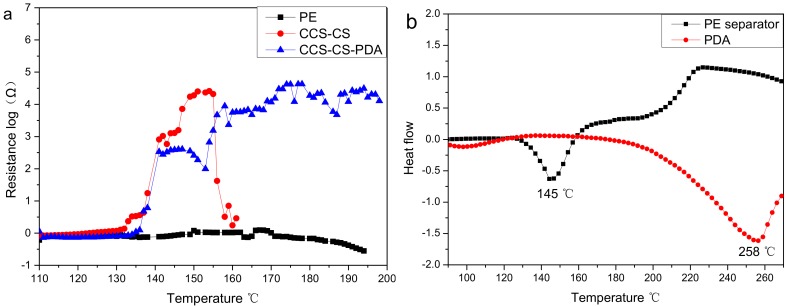
(**a**) The shutdown behavior of the separator; (**b**) DSC of the PE separator and PDA.

The morphology and porosity change of the separator at high temperature suggest that the pores inside the PE separator will collapse at high temperature. Cells will fail as the ion conduction is cut off, before large thermal shrinkage of the separator occurs and leads to the cell internal shorting, which will play an important self-protection ability (shutdown function) contributed by the separator. The shutdown behavior of a separator was determined by electrochemical impedance measurement with temperature increment at a rate of 1 °C·min^–1^ as shown in Figure 5a. It can be found from the figure that the PE separator exhibited no obvious change in impedance. The impedance change with the temperature of the PE separator was studied separately as shown in Figure S2. The internal resistance of the PE separator exhibited two weak declines in the peaks at 105 and 132 °C, which corresponded to the initial shrinking temperature and the PE materials’ melt temperature, respectively. The volatility in the impedance of the separator above 140 °C may be caused by the acute shrink movement of the PE separator. Both CCS-CS and CCS-CS-PDA exhibited a sharp increase in the internal resistance at approximately 138 °C. The melted PE material blocked the hole of the CCS-CS and CCS-CS-PDA, and ionic transport between the electrodes was effectively stopped, which resulted in the above impedance change. However, the impedance of CCS-CS decreased to almost 0 Ω at 160 °C, which suggested that the CCS-CS suffered obvious dimensional change; thus, an electrical short circuiting of the battery was unavoidable as the cathode and anode can no longer be separated by the separator. On the contrary, the resistance of CCS-CS-PDA still stayed at high values even above 200 °C. The impedance change indicates that the CCS-CS-PDA can stay stable at high temperatures. 

To clarify the change in impedance with the temperature of the separator, the differential scanning calorimetry (DSC) measurements of the PE separator and PDA were carried out and are shown in Figure 5b. From the figure, we observed that the PE separator had one endothermic peak at near 145 °C, which could be attributed to the melting of the high molecular weight polyethylene materials. The DSC profiles agreed well with the above impedance increase of the CCS-CS and CCS-CS-PDA. The DSC profiles of PDA shown in Figure 5b only exhibited an endothermic peak at about 258 °C. The improvement in thermal stability of the CCS-CS-PDA can be attributed to the application of high temperature resistance PDA.

**Figure 6:**
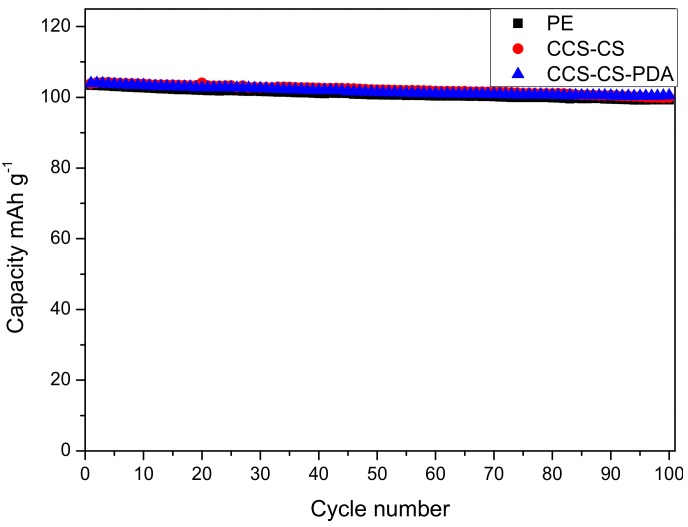
Cycle performance of coin cells with the PE separator, CCS-CS and CCS-CS-PDA.

**Figure 7:**
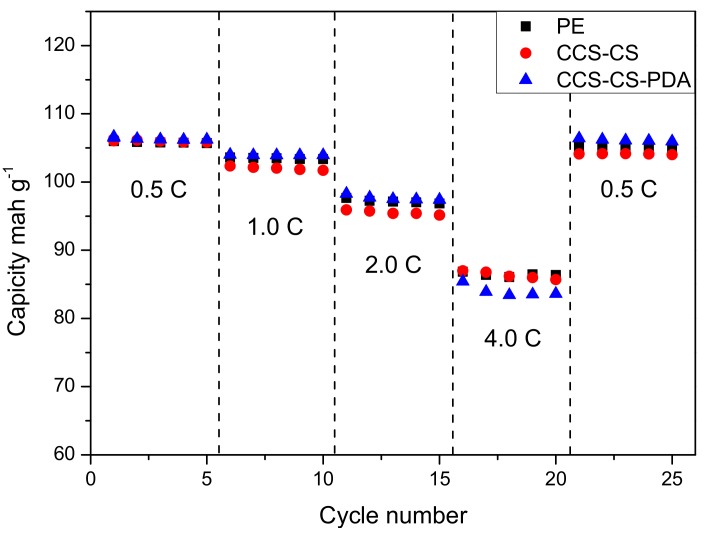
Rate performance of the batteries with the PE separator, CCS-CS and CCS-CS-PDA.

To examine the effect of PDA coating layer on the electrochemical performance, the cyclic performance and rate capability of cells using coin-type half cells based on the Li anode and the LiMn_2_O_4_ cathode were tested, and the results are shown in Figure 6 and Figure 7. Cells were charged and discharged for 100 cycles from 3.0 to 4.2 V at a 1 C rate for the cyclic performance test. The discharge capacities of coin-type cells with the PE separator, CCS-CS-PDA and CCS-CS separator were very close as shown in Figure 6. Figure 7 shows the rate performance of the coin cells prepared with the PE separator, CCS-CS and CCS-CS-PDA. Cells were charged to 4.2 V and discharged to 3 V at the current rates of 0.5, 1.0, 2.0, 4.0 and 0.5 C, respectively. The discharge capacities of cells with different separators decreased gradually with the increase in rate and were almost similar at 0.5 and 1.0 C (low rate). Compared to the cells assembled with the PE separator, the CCS-CS separator and the CCS-CS-PDA showed slightly lower rate capabilities at 2.0 and 4.0 C (high rate). The result is well accordant with the result of ionic conductivity.

## 4. Conclusions

In this study, the CCS-CS prepared with the PE separator, Al_2_O_3_ inorganic particles and CMC and SBR mix binders were further modified by coating with a thin PDA layer. Compared with pure CCS-CS, the CCS-CS-PDA exhibits excellent thermal stability, which shows no thermal shrinkage after being stored at 200 °C for 30 min. Compared with the pure PE separator, the uptake and wettability with the electrolyte and water of CCS-CS-PDA are improved significantly. Furthermore, the test of electrochemical impedances changing with the temperature suggests that the CCS-CS-PDA shows a shutdown temperature range from 138 to more than 200 °C. The cells prepared with the CCS-CS-PDA also show stable repeated cycling performance and rate capacity at room temperature. Such advantages of CCS-CS-PDA mentioned above make it suitable for applications in secondary lithium batteries, especially in the energy storage system and HEV areas.

## Figures and Tables

**Table 1:** The electrolyte uptake and ionic conductivity of the separator.

Separator	PE separator	CCS-CS	CCS-CS-PDA
Weight mg	3.1	4.3	4.6 ± 0.1
Porosity %	41.5 ± 0.5	41.2 ± 0.5	35.3 ± 0.5
Average uptake %	54 ± 1	71.2 ± 2	70.3 ± 2
Contact angle with electrolyte	35	0	0
AC impedance mS·cm^–1^	0.78 ± 0.01	1.10 ± 0.01	0.71 ± 0.01

## Supplementary Materials

The following are available online at www.mdpi.com/2073-4360/9/5/159/s1: Figure S1: Porosity change with the temperature of the PE separator, CCS-CS and CCS-CS-PDA; Figure S2: Shutdown behavior of the PE separator.
