# Supplementary material for: A Modified Ceramic-Coating Separator with High-Temperature Stability for Lithium-Ion Battery
Source: Polymers (Basel). 2017 Apr 29;9(5):159. doi: 10.3390/polym9050159 (PMC6432417; doi:10.3390/polym9050159)
Supplement: Supplementary file 1 [file polymers-09-00159-s001.pdf]

Supplementary Materials: A Modified Ceramic-Coating Separator with High-Temperature Stability for Lithium-Ion Battery

Chuan Shi, Jianhui Dai, Chao Li, Xiu Shen, Longqing Peng, Peng Zhang, Dezhi Wu, Daoheng Sun and Jinbao Zhao

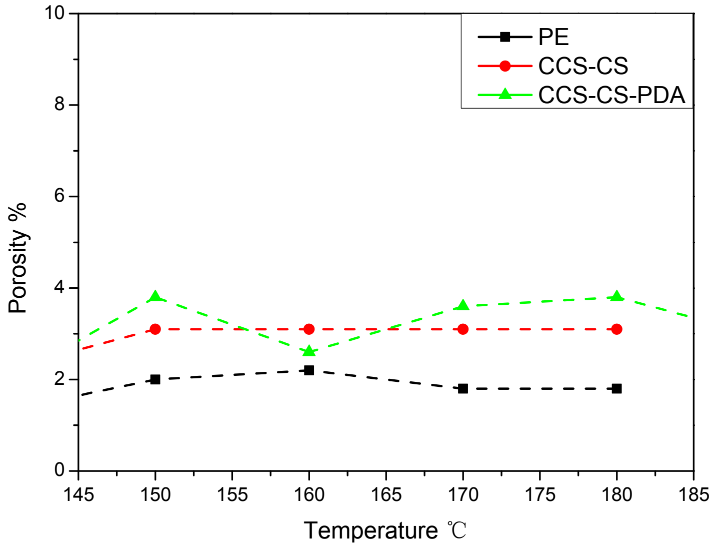

Figure S1. Porosity change with the temperature of the PE separator, CCS-CS and CCS-CS-PDA.

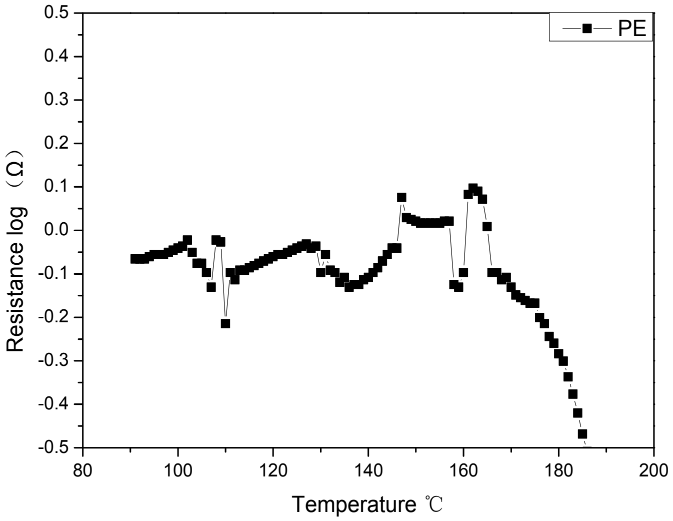

Figure S2. Shutdown behavior of the PE separator.
